# *PPE* Barcoding Identifies Biclonal Mycobacterium ulcerans Buruli Ulcer, Côte d’Ivoire

**DOI:** 10.1128/spectrum.00342-23

**Published:** 2023-05-24

**Authors:** B. G. O. Tchan, S. Ngazoa-Kakou, N. Aka, N. K. B. Apia, N. Hammoudi, M. Drancourt, J. Saad

**Affiliations:** a IRD, MEPHI, IHU Méditerranée Infection, Aix-Marseille-Université, Marseille, France; b IHU Méditerranée Infection, Marseille, France; c Plateforme de Biologie Moléculaire, Institut Pasteur de Côte d'Ivoire, Abidjan, Côte d'Ivoire; d Unité des Mycobactéries Tuberculeuses et Atypiques, Institut Pasteur de Côte d'Ivoire, Abidjan, Côte d'Ivoire; University Paris-Saclay, AP-HP Hôpital Antoine Béclère, Service de Microbiologie, Institute for Integrative Biology of the Cell (I2BC), CEA, CNRS

**Keywords:** *Mycobacterium ulcerans*, *Mycobacterium marinum*, *PPE* gene, genotyping, whole-genome sequencing, Buruli ulcer

## Abstract

Mycobacterium ulcerans, an environmental opportunistic pathogen, causes necrotic cutaneous and subcutaneous lesions, named Buruli ulcers, in tropical countries. PCR-derived tests used to detect M. ulcerans in environmental and clinical samples do not allow one-shot detection, identification, and typing of M. ulcerans among closely related Mycobacterium marinum complex mycobacteria. We established a 385-member M. marinum/M. ulcerans complex whole-genome sequence database by assembling and annotating 341 M. marinum/M. ulcerans complex genomes and added 44 M. marinum/M. ulcerans complex whole-genome sequences already deposited in the NCBI database. Pangenome, core genome, and single-nucleotide polymorphism (SNP) distance-based comparisons sorted the 385 strains into 10 M. ulcerans taxa and 13 M. marinum taxa, correlating with the geographic origin of strains. Aligning conserved genes identified one *PPE* (proline-proline-glutamate) gene sequence to be species and intraspecies specific, thereby genotyping the 23 M. marinum/M. ulcerans complex taxa. PCR sequencing of the *PPE* gene correctly genotyped nine M. marinum/M. ulcerans complex isolates among one M. marinum taxon and three M. ulcerans taxa in the African taxon (T2.4). Further, successful *PPE* gene PCR sequencing in 15/21 (71.4%) swabs collected from suspected Buruli ulcer lesions in Côte d’Ivoire exhibited positive M. ulcerans *IS*2404 real-time PCR and identified the M. ulcerans T2.4.1 genotype in eight swabs and M. ulcerans T2.4.1/T2.4.2 mixed genotypes in seven swabs. *PPE* gene sequencing could be used as a proxy for whole-genome sequencing for the one-shot detection, identification, and typing of clinical M. ulcerans strains, offering an unprecedented tool for identifying M. ulcerans mixed infections.

**IMPORTANCE** We describe a new targeted sequencing approach that characterizes the *PPE* gene to disclose the simultaneous presence of different variants of a single pathogenic microorganism. This approach has direct implications on the understanding of pathogen diversity and natural history and potential therapeutic implications when dealing with obligate and opportunistic pathogens, such as Mycobacterium ulcerans presented here as a prototype.

## INTRODUCTION

Mycobacterium ulcerans, a nontuberculous mycobacterium aggregated in a so-called Mycobacterium marinum/M. ulcerans complex, is responsible for Buruli ulcer, a neglected tropical infection that occurs in rural populations in 36 countries and 4 continents (1). M. ulcerans isolates have been generated from clinical samples of Buruli ulcer lesions (2), in contrast to only one environmental M. ulcerans strain 00-1441 confirmed after a 3-month-long isolation in Bactec culture followed by mouse footpad inoculation from one *Gerris* sp. in Ghana (3). This M. ulcerans strain, however, is not available from any public microorganism collection, and its genome sequence is not known, limiting any further study of this unique environmental isolate.

The detection of M. ulcerans is routinely based on the detection of nucleic acid sequences thought to be specific for this opportunistic pathogen. More precisely, detection of M. ulcerans in samples mainly relies on PCR-based multiplex detection of the plasmid-encoded Ketereductase B-domain gene (KR-B) and two insertion sequences *IS*2404 and *IS*2606 (4). This multiplex system is limited in its capability to accurately identify M. ulcerans from closely related mycobacteria, including M. ulcerans subsp. *shinshuense* and Mycobacterium liflandii, and to genotype M. ulcerans in a context where the geographical microevolution of the pathogen has been observed in several Buruli ulcer regions of endemicity in Africa and Australia (5).

Here, investigating a large collection of M. marinum/M. ulcerans complex whole-genome sequences (WGSs) in the context of taxonomic and phylogenetic investigations of this pathogen (6), we observed one hot spot of genomic sequence variation among M. ulcerans complex members (including different taxa). We further analyzed this hot spot and are now reporting that investigating this hot spot by using an appropriate PCR sequencing system described here could add a convenient laboratory tool for diagnosing genomic diversity of M. ulcerans in clinical materials.

## RESULTS

### WGS database.

We investigated a database containing a total of 385 M. marinum complex genomic sequences. In detail, 355 M. ulcerans genomes were isolated from humans and animals from Angola, Benin, Cameroon, Côte d'Ivoire, Democratic Republic of the Congo, Gabon, Ghana, Nigeria, Papua New Guinea, Republic of the Congo, Togo, Uganda, Australia (Mornington Peninsula, Bellarine Peninsula, Gippsland, Melbourne, Ballarat, Phillip Island, Queensland, Darwin, and Port Hedland regions), the United States, Japan, and French Guiana, 3 *M. pseudoshottsii* genomes were isolated from the United States and China, 3 *M. liflandii* genomes were isolated from Israel, and 24 M. marinum genomes were isolated from France, Thailand, Israel, the United States, Japan, and the United Kingdom.

### WGS analyses.

**FIG 1:**
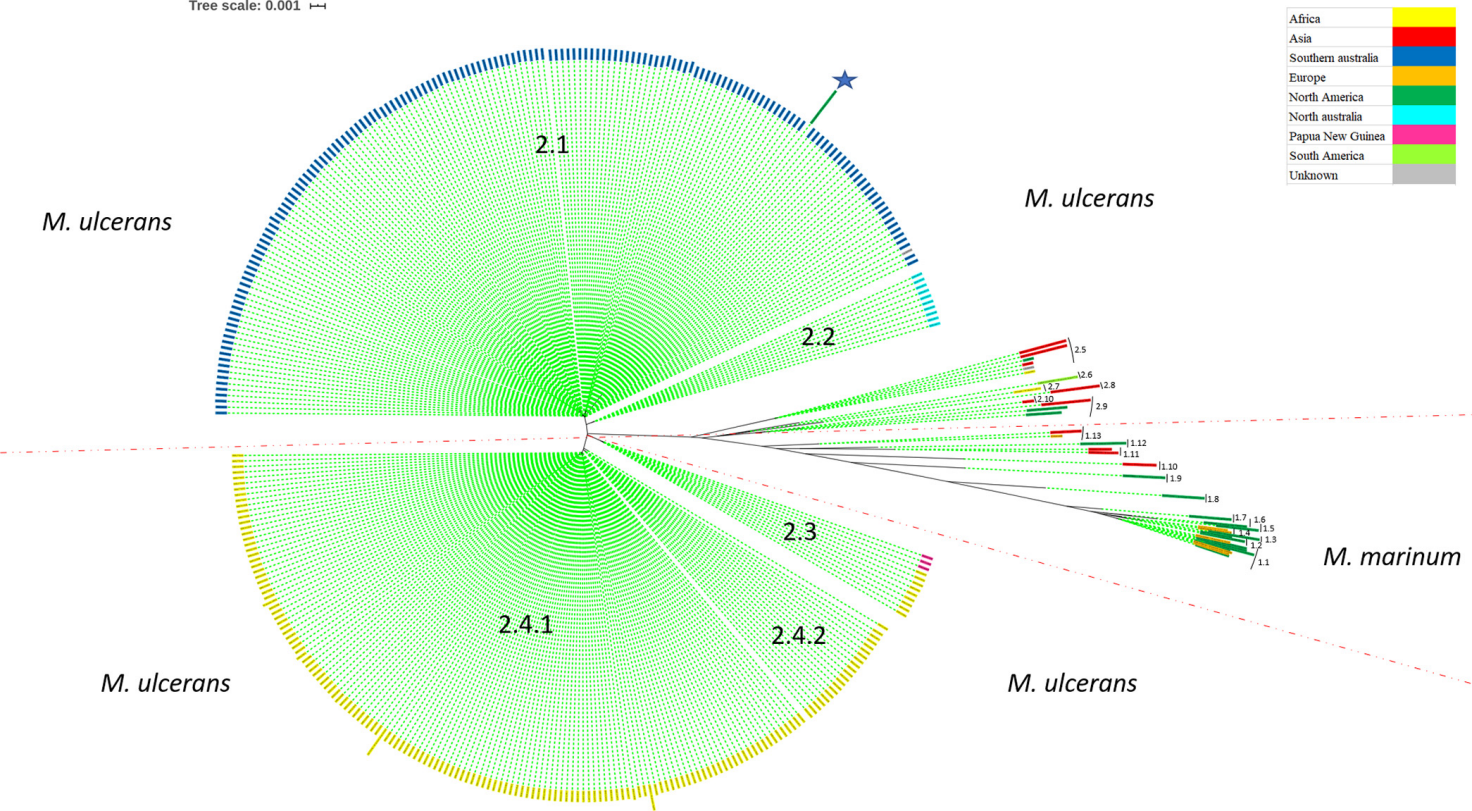
Phylogenetic tree of 385 strains of the M. marinum/M. ulcerans complex showing the different detailed taxa. An unclassified strain is represented by a blue star. FastTree v2.1.11 on the NGphylogeny website was used to create a phylogenetic tree using default parameters.

**FIG 2:**
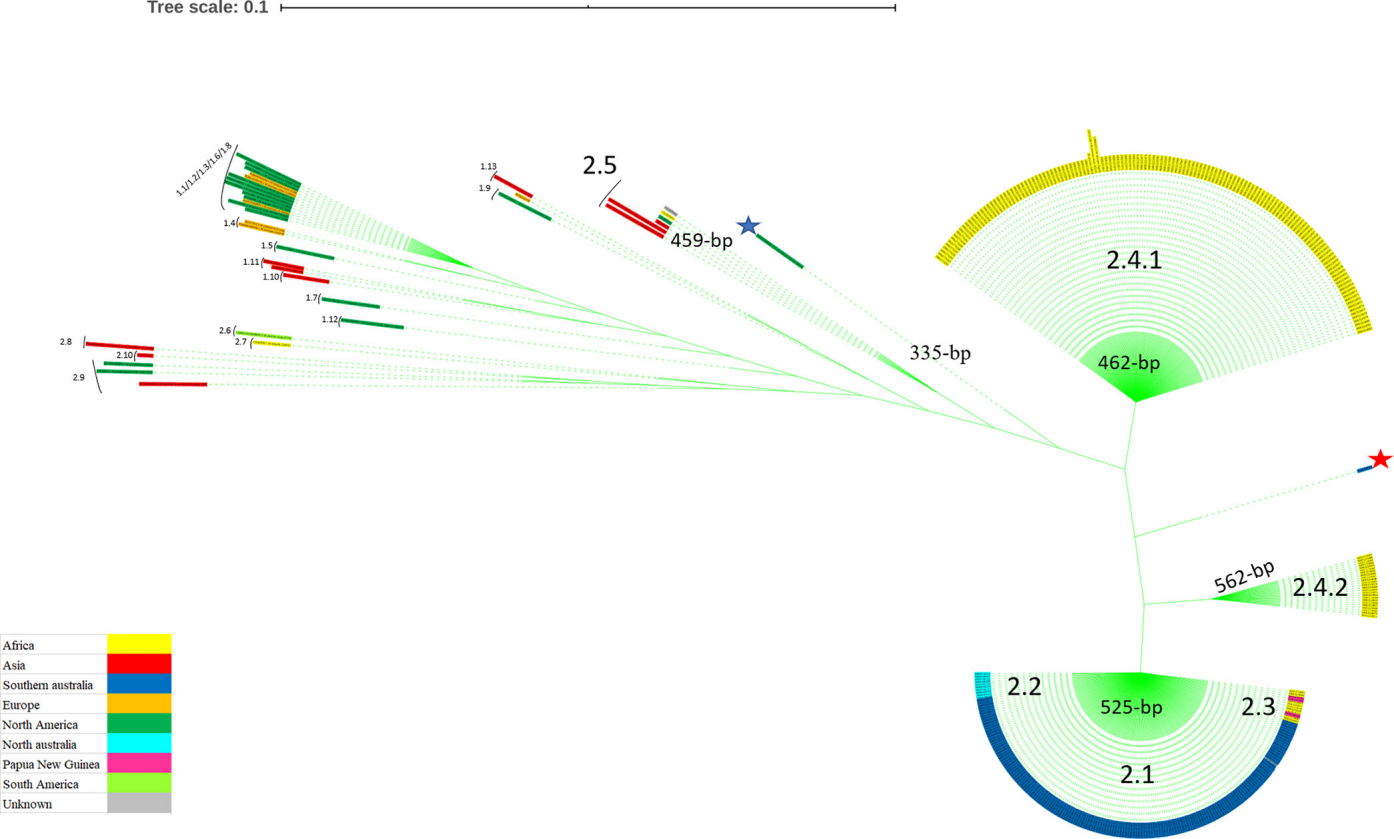
Phylogenetic tree of the *PPE* (proline-proline-glutamate; GenBank *PPE* gene ID for Mycobacterium ulcerans Agy99: ABL03116.1) gene extracted from 384 genomes of strains of M. ulcerans and M. marinum clades showing the different characterized taxa. An unclassified strain is represented by a blue star, and the SRR6346364 isolate is represented by a red star. FastTree v2.1.11 on the NGphylogeny website was used to create a phylogenetic tree using default parameters.

**FIG 3:**
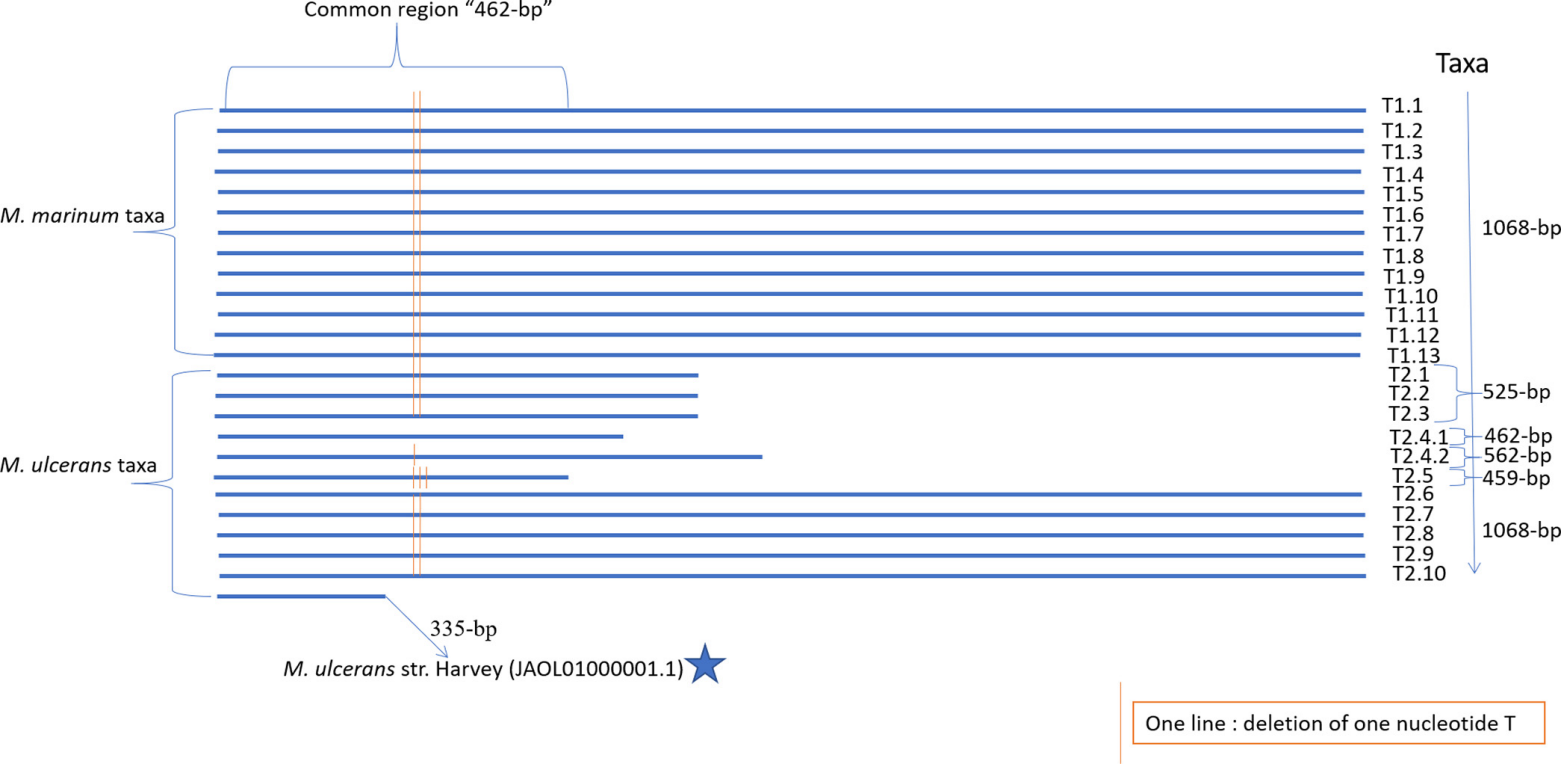
Alignment profile of the extracted *PPE* (proline-proline-glutamate; GenBank *PPE* gene ID for Mycobacterium ulcerans Agy99: ABL03116.1) gene from genomes showing the different sizes of this gene based on taxa of M. ulcerans and M. marinum.

Pangenome analysis of the above-listed 385 genomes (Table S1 in the supplemental material) yielded a total of 54,211 genes comprising 2,804 core genes (99% ≤ strains ≤ 100%), 783 soft core genes (95% ≤ strains < 99%), 2,155 shell genes (15% ≤ strains < 95%), and 48,469 cloud genes (0% ≤ strains < 15%). Core genome tree and single-nucleotide polymorphism (SNP) distance based on core genome sequence data (2,617,970 bp) yielded 10 different M. ulcerans taxa and 13 M. marinum taxa comprising 24 isolates, as previously reported (Fig. 1; Tables S1 and S2) (7). In detail, the 13-member M. marinum taxa (1) was composed of M. marinum taxon 1.1, which includes 8 isolates with between 5 and 767 SNPs, M. marinum taxon 1.2, which includes 2 isolates with 13 SNPs, M. marinum taxon 1.3, which includes 1 isolate, M. marinum taxon 1.4, which includes 2 isolates with 18 SNPs, M. marinum taxon 1.5, which includes 1 isolate, M. marinum taxon 1.6, which includes 1 isolate, M. marinum taxon 1.7, which includes 1 isolate, M. marinum taxon 1.8, which includes 1 isolate, M. marinum taxon 1.9, which includes 1 isolate, M. marinum taxon 1.10, which includes 1 isolate, M. marinum taxon 1.11, which includes 2 isolates with 2 SNPs, M. marinum taxon 1.12, which includes 1 isolate, and M. marinum taxon 1.13, which includes 2 isolates with 88 SNPs (Fig. 1; Table S2). As for M. ulcerans, the M. ulcerans taxon (2.1) was composed of 165 isolates all from Southern Australia, with SNPs varying between 1 and 618 (including isolates originating from six counties [that is, Ballarat, Mornington Peninsula, Phillip Island, Melbourne, Gippsland, and Bellarine Peninsula]), and 6 animal isolates (1 Canis lupus familiaris, 3 *Trichosurus vulpecula*, 1 “koala,” and 1 *Potorous longipes*) originating from four counties (that is, Mornington Peninsula, Phillip Island, Gippsland, and Bellarine Peninsula). The M. ulcerans taxon 2.2 was comprised of 10 isolates exclusively from North Australia, with SNPs varying between 1 and 395 (including 1 isolate from Darwin, 1 from Port Hedland, and 8 from Queensland). *M. ulcerans* taxon 2.3 was detected in Africa and North Australia; Papua New Guinea included 12 isolates with SNPs varying between 7 and 489 (including 5 isolates from Africa, Benin [5], Côte d’Ivoire [1], Cameroon [1], and Gabon [2], and North Australia, Papua New Guinea [3]). M. ulcerans taxon 2.4 (specific for Africa) included 160 isolates with SNPs varying between 1 and 955 (in total, including isolates originating from 11 Africa countries, that is, Angola, Benin, Cameroon, Côte d'Ivoire, Democratic Republic of the Congo, Gabon, Ghana, Nigeria, Republic of the Congo, Togo, and Uganda). Taxon 2.4 was divided into subtaxon 2.4.1, which included 139 isolates with SNPs varying between 1 and 655, and subtaxon 2.4.2, which included 21 isolates with SNPs varying between 4 and 598. M. ulcerans taxon 2.5 included 6 isolates, with SNPs varying between 9 and 256 (including 1 isolate of unknown origin, 3 from Japan, 1 from the United States, and 1 from Uganda). M. ulcerans taxon 2.6 (specific for French Guiana) included one isolate (M. ulcerans P7741 isolated from French Guiana). Taxon 2.7 included *M. liflandii* 128FXT isolated in Africa. Taxon 2.8 included M. marinum strain BB170200 isolated in Israel. Taxon 2.9 included 3 isolates with SNPs varying between 254 and 509 (*M. pseudoshottsii JCM 15466*, *M. pseudoshottsii*, and M. marinum strain DL240490). Taxon 2.10 included Mycobacterium sp. 012931. Interestingly, we found the M. ulcerans strain Harvey (JAOL01000001.1) with 1,363 SNPs within taxon 2.1. This strain has remained unclassified and is waiting for genome sequence confirmation to ensure the correct classification of this atypical strain (Fig. 1 to 3, blue star; Table S2).

### Identification of taxa using the *PPE* gene as a hot spot-specific target.

**FIG 4:**
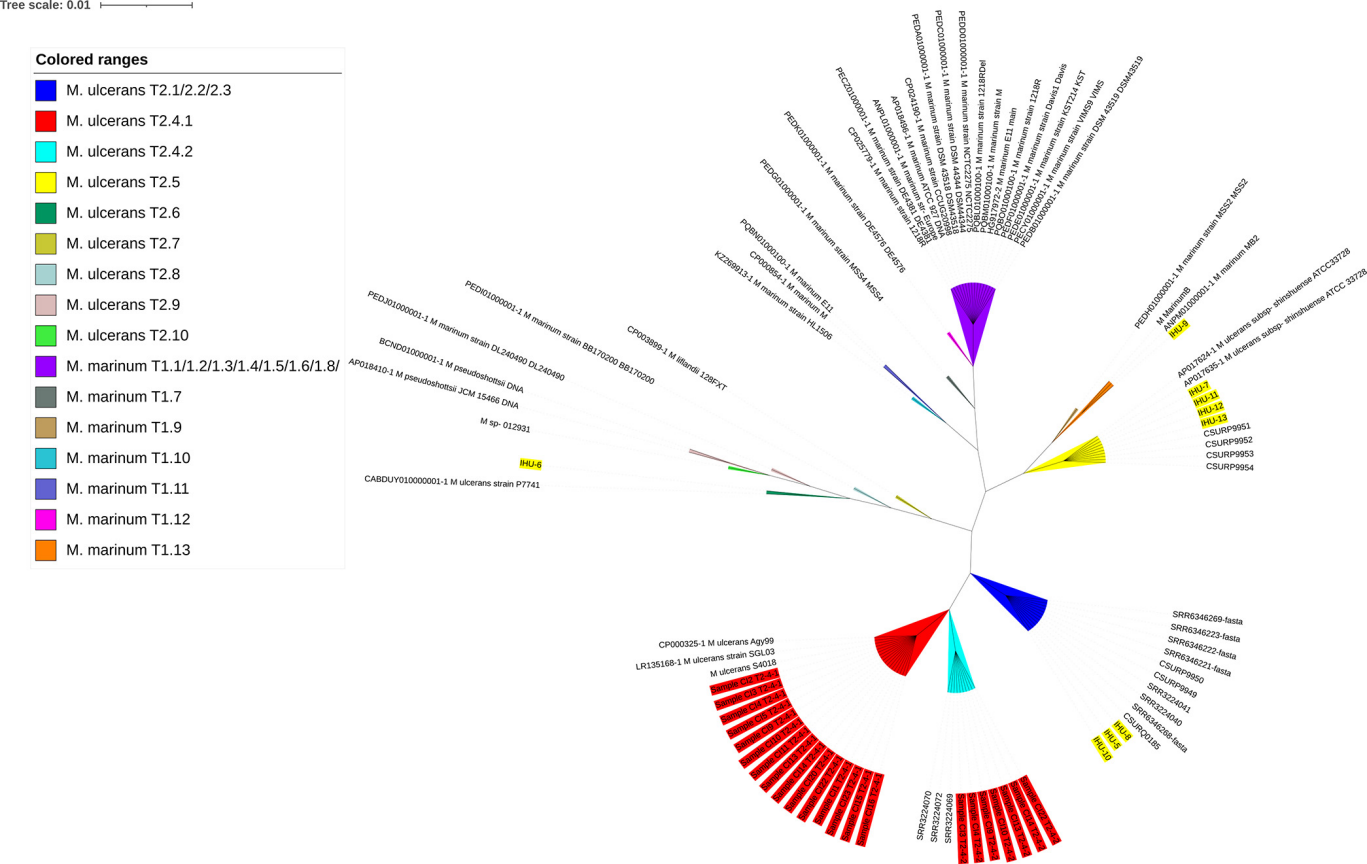
Partial *PPE* (proline-proline-glutamate; GenBank *PPE* gene ID for Mycobacterium ulcerans Agy99: ABL03116.1) gene sequence-based phylogenetic tree of 9 IHU clinical strains (IHU 5 to 13) and 15 clinical samples from Côte d'Ivoire (CI) with the extracted *PPE* gene from genomes of several strains as the reference for each taxon; background label yellow: IHU5 = CSURQ0185, IHU6 = CSURP7741, IHU7 = CSURP9954, IHU8 = CSURP9950, IHU9 = M. marinum B, IHU10 = CSURP9949, IHU11 = CSURP9951, IHU12 = CSURP9952, S13 = CSURP9953; background label red: CI. FastTree v2.1.11 on the NGphylogeny website was used to create a phylogenetic tree using defaults parameters.

Based on output conserved alignment genes from the pangenome analysis of 385 different M. ulcerans and M. marinum isolates from different areas, we searched for a specific marker gene whose sequence would detect and discriminate between the announced taxa. Using the Seaview application, we observed that one *PPE* gene sequence was variable enough to discriminate between the M. ulcerans and M. marinum taxa, acting as a hot spot sequence proxy for the complete genome sequence, as detailed above. M. ulcerans strain SRR6346343 in taxon 2.1, isolated from *T. vulpecula* in Gippsland, completely lacked the *PPE* gene. A phylogenetic tree drawn from 384 extracted *PPE* gene sequences in the assembled genomes of M. marinum and M. ulcerans showed coherent clustering of taxa with the core genome phylogenetic tree (Fig. 2). This 1,068-bp *PPE* gene was annotated as a PPE family protein (CP003899.1) in the *M. liflandii* strain 128FXT used as the reference (GenBank accession number NC_020133.1). The PPE protein was predicted to be an alpha helical transmembrane protein with a 0.934205 probability. We further observed deletions in the 5′ extremity of the *PPE* gene detected specifically in M. ulcerans taxa 2.1, 2.2, 2.3, 2.4.1, and 2.4.2 and the M. ulcerans strain Harvey (JAOL01000001.1), resulting in a *PPE* gene size varying between 335 bp and 562 bp, correlating with the clustering of taxa in the core genome tree (Fig. 3). In M. ulcerans taxon 2, these deletions resulted in the prediction of a nontransmembrane protein with a 0.90177 probability. As a result, the seven truncated *PPE* genes are classified as pseudogenes. Consequently, the complete *PPE* gene phylogenetic tree showed distinct discrimination between all M. ulcerans and M. marinum taxa, except a lack of difference between M. marinum taxa 1.1, 1.2, 1.3, 1.6, and 1.8 (Fig. 2). Aligning 383 *PPE* gene sequences showed a common 462-bp region, including several specific mutations and local deletions that discriminated between M. ulcerans and M. marinum taxa, except a lack of difference between Australian M. ulcerans taxa 2.1, 2.2, and 2.3 and M. marinum taxa 1.1, 1.2, 1.3, 1.4, 1.5, 1.6, and 1.8 (Fig. 3 and 4). All the mutations or deletions in this region were noted in each taxon according to their position (Table S3). M. ulcerans taxa 2.1, 2.2, and 2.3 were characterized by two TT deletions in the 378 to 379 sequence position. Subtaxon 2.4.1 lacked a deletion at this position. Subtaxon 2.4.2 was characterized by one T deletion at position 378, and taxon 2.5 was characterized by three TTT deletions at positions 378 to 380 (Fig. 3; Table S3). In taxon 2.1, the Australian isolate SRR6346364 has two unique mutations (C/G at position 66 and C/A at position 86) in the common region compared to all the other isolates (Fig. 2, red star). In conclusion, here, pangenome analysis of 385 M. marinum/M. ulcerans complex genomes indicated a geographic clustering of isolates. Specifically for M. ulcerans, we detailed two taxa specific for Australia (T2.1/T2.2), two taxa specific for Africa (T2.4.1/T2.4.2), one taxon (T2.5) specific for Asia, and one taxon (T2.3) shared between Africa and Papua New Guinea.

### Experimental data.

Partial PCR sequencing of the *PPE* gene in nine cultured M. ulcerans and M. marinum isolates belonging to four taxa retrieved an experimental sequence identical to the one derived *in silico* from the whole-genome sequence of the same strain (Fig. 4). Regarding the mutation profile, we detected the same mutation profile in each isolate as the standard mutation *PPE* gene for each strain (Table S3). As for DNA extracted from 24 swabs collected for the diagnosis of Buruli ulcers, the insertion sequence *IS*2404 real-time PCR found 21 positive samples exhibiting cycling threshold (*C_T_*) values of <32 (Table 1). PCR of the *PPE* gene further detected 15/21 (71.4%) positive samples, which all exhibited real-time PCR *IS*2404 *C_T_* values of <39.9 (Fig. 4; Table 1). Sequencing PCR products showed that the 15 samples were related to Africa taxon T2.4 (Fig. 4). In detail, the M. ulcerans T2.4.1 genotype was detected in eight samples, whereas a biclonal T2.4.1/T2.4.2 genotype infection was observed in seven samples by detecting the specific character of each taxon on output sequencing reads. Interestingly, biclonal infection was observed specifically in Toumodi and Sakassou regions, two cities located 15 miles apart one from the other, suggesting a cluster of cases in this region. The consensus sequence of each taxon was extracted, and a phylogenetic tree was generated with the reference *PPE* gene (Fig. 4).

**TABLE 1:** Results from real-time PCR of *IS*2404 and standard PCR (PCRst) of the *PPE* gene in 24 clinical Buruli ulcer samples isolated from Côte d'Ivoire

Samples	PCR *IS*2404 (*C_T_*)^*a*^	PCRst *PPE*
CI5	22.5	Positive
CI3	22.64	Positive
CI14	25.25	Positive
CI13	25.3	Positive
CI22	25.66	Positive
CI10	26.82	Positive
CI20	27.23	Positive
CI9	27.93	Positive
CI1	28.02	Positive
CI4	28.14	Positive
CI23	28.66	Positive
CI2	29.1	Positive
CI15	31.38	Positive
CI16	31.53	Positive
CI11	31.9	Positive
CI18	31.92	Negative
CI21	32.07	Negative
CI8	33.02	Negative
CI17	34.67	Negative
CI7	35.06	Negative
CI24	35.58	Negative
CI6	NA	Negative
CI19	NA	Negative
CI25	NA	Negative

a NA, not available.

## DISCUSSION

To complement public databases currently containing only 14 complete M. ulcerans genome sequences, here, we succeeded in assembling a total of 341 additional complete M. marinum/M. ulcerans complex genome sequences directly from reads recovered from isolates acquired from two large epidemic areas, Africa and Australia. This work gave us the opportunity to conduct an unprecedented pangenome comparative analysis of many M. marinum/M. ulcerans complex members, with proposals for the reclassification of M. marinum/M. ulcerans complex members in different taxa based on core genomes, SNP distance, and geographical isolation of isolates, following our previous preliminary study.

As a practical output for the detection of M. marinum/M. ulcerans complex organisms in clinical practice, this tremendous bioinformatic work indicated that one *PPE* gene was a hot spot for genome variability. *In silico* data indicated that a 390-bp chromosomal fragment sequence of the *PPE* gene discriminated between the different taxa regions derived from a previous WGS analysis (5–8) and was experimentally confirmed on cultured samples and on clinical samples in the presence of negative controls. Together, these data indicate that the PCR-sequencing protocol described here could be used for the effective one-shot detection, identification, and genotyping of organisms within the M. marinum/M. ulcerans complex in clinical samples previously detected by *IS*2404 real-time PCR. In the present study, negative detection of the *PPE* gene in some clinical samples correlated with low specific DNA concentration, marked by *IS*2404 real-time PCR *C_T_* values of >32. Indeed, whereas the number of *IS*2404 copies varies from 150 to 200 copies in the M. ulcerans genome (4, 9), the *PPE* gene investigated here is a monocopy gene. Moreover, the *PPE* gene PCR sequencing system developed here may miss *PPE* gene deletions, making it undetectable by *PPE* gene primers.

The protocol developed here could be of interest for laboratories investigating M. marinum/M. ulcerans complex organisms in tropical countries, as M. ulcerans and M. marinum genotyping is currently achieved by analyzing mycobacterial interspersed repetitive units (9). The only obvious alternative would be whole-genome sequencing, an approach so far limited to M. marinum/M. ulcerans complex isolates, as no metagenomic data have ever been reported directly from Buruli ulcer lesions; therefore, the actual genomic diversity of M. ulcerans that causes Buruli ulcers remains underestimated. The protocol reported here therefore offers a convenient, albeit limited, proxy for the study of polyclonal M. ulcerans infection. Accordingly, we report a biclonal M. ulcerans infection in Buruli ulcer lesions in Côte d’Ivoire, an unprecedented observation opening new avenues in terms of understanding the natural history of Buruli ulcers in front of a yet undetermined genomic diversity of environmental M. ulcerans.

We therefore propose that partial *PPE* gene PCR sequencing could be implemented in the routine laboratory investigation of otherwise PCR-confirmed Buruli ulcer lesions to assess polyclonal infection. The protocol reported here will be adapted to the rapid Oxford Nanopore sequencing platform, which is based on a mapping approach, for point-of-care application.

## MATERIALS AND METHODS

### *In silico* M. ulcerans whole-genome sequence analyses.

We retrieved WGS data for 44 assembled genomes of M. ulcerans and M. marinum available in the NCBI database (parameter used: Assembly; November 2020) as well as reads deposited for 174 M. ulcerans and 167 M. ulcerans isolates from two large epidemic areas in Australia and Africa (Table S1 in the supplemental material) (5–8). FastQC was used for quality control of the sequencing data (https://github.com/s-andrews/FastQC). These 341 M. ulcerans sequencing reads were assembled using SPAdes version 3.14.0 and annotated using Prokka version 1.14.6 (10, 11). To run SPAdes, we used the pipeline option “–careful” to reduce the number of mismatches and short insertions/deletions (indels). Default parameters for *k* values, that is, *k*-mer values of 127, 99, 77, 55, 33, and 21, were applied. All contigs with sizes under 800 bp or with depth values lower than 25% of the mean depth were possible contaminants and were removed. Roary 3.13.0 set at 95% sequence identity was used to generate the pangenome and core genome (conserved genes) (12). Snp-dists 0.6.3 was then used to calculate the single-nucleotide polymorphism (SNP) distance based on the core genome of the 385 studied WGSs. The core genome alignment was visualized using Seaview (13). FastTree v2.1.11 on the NGphylogeny website was used to create a phylogenetic tree using default parameters of the core genome and of the *PPE* gene of interest in this study (M. ulcerans Agy99; GenBank *PPE* gene ID: ABL03116.1). Moreover, one PCR primer pair specifically targeting a 390-bp region in the *PPE* gene of interest was designed using primer BLAST (https://www.ncbi.nlm.nih.gov/tools/primer-blast/).

### *PPE* gene-based PCR.

Eight M. ulcerans isolates and one M. marinum isolate maintained in the IHU Méditerranée Infection Mycobacteriology Laboratory collection and firmly identified by WGS were incubated for 6 weeks at 30°C on solid Löwenstein-Jensen medium (Bio-Rad, Marnes la Coquette, France). DNA was extracted from colonies using the InstaGene matrix following the manufacturer’s instructions (Bio-Rad). In addition, 24 leftover swab samples taken from suspected Buruli ulcer cutaneous lesions in 24 different patients as part of routine medical management in Côte d'Ivoire were tentatively diagnosed as M. ulcerans infections at the Institut Pasteur Laboratory, Abidjan, Côte d’Ivoire (Table 1). Swabs were hydrated and suspended in sterile distilled water, which was used for the extraction of total DNA using a Qiagen kit following the manufacturer’s instructions (DNeasy blood and tissue kit, Qiagen, Courtaboeuf, France). M. ulcerans DNA was searched using a real-time PCR targeting the *IS*2404 gene, as previously described (4). After strict anonymization of leftover extracted DNA, the remaining DNA was sent to IHU Méditerranée Infection to refine the diagnosis using the *PPE* gene and was stored at −20°C until use. PCR amplifications of a 429-bp fragment were performed in a Bio-Rad MyCycler (Bio-Rad, Marnes-la-Coquette, France) in 50-μL reaction mixtures containing 5 μL of genomic DNA template, 25 μL of AmpliTaq Gold 360 master mix (Thermo Fisher Scientific), 1.5 μL of each primer (25 μM stock; forward primer: 5′-GGCGTCTGTTTGGATGGCCT-3′ [positions 413654 to 413672, M. ulcerans Agy99 reference, GenBank: ABL03116.1]; reverse primer: 5′-CCGCGCGTAGTCGACTTCG-3′ [positions 414062 to 414082, M. ulcerans Agy99 reference, GenBank: ABL03116.1]), and 17 μL of deionized water. The PCR program included an initial denaturation at 95°C for 15 min, then 35 cycles of 95°C for 30 s, 64°C for 30 s, and 72°C for 1 min 30 s, followed by a final extension at 72°C for 10 min. DNA from a Mycobacterium bovis BCG strain treated under the same conditions was used as a negative control. Amplification products, confirmed by 1.5% agarose gel electrophoresis, were purified using the Millipore NucleoFast 96 PCR kit according to the manufacturer’s recommendations (Macherey-Nagel, Düren, Germany) and sequenced using the BigDye terminator cycle sequencing kit (Applied Biosystems) with an ABI automated sequencer (Applied Biosystems). Sequences were assembled using the Chromas Pro 1.7 software (Technelysium Pty, Ltd., Tewantin, Australia).

### Ethics statements.

No clinical specimen was sampled specifically for this study, which only incorporated anonymized, leftovers of samples routinely collected for the diagnosis of Buruli ulcers in consulting patients.

### Data availability.

The data supporting the findings of this study are available within the article and its supplemental material. Raw data that support the findings of this study are available from the corresponding author upon reasonable request.

## References

[1] Zingue D, Bouam A, Tian RBD, Drancourt M. 2018. Buruli ulcer, a prototype for ecosystem-related infection, caused by *Mycobacterium ulcerans*. Clin Microbiol Rev 31:e00045-17. doi:10.1128/CMR.00045-17. 29237707 PMC5740976

[2] Van Leuvenhaege C, Vandelannoote K, Affolabi D, Portaels F, Sopoh G, de Jong BC, Eddyani M, Meehan CJ. 2017. Bacterial diversity in Buruli ulcer skin lesions: challenges in the clinical microbiome analysis of a skin disease. PLoS One 12:e0181994. doi:10.1371/journal.pone.0181994. 28750103 PMC5531519

[3] Portaels F, Meyers WM, Ablordey A, Castro AG, Chemlal K, de Rijk P, Elsen P, Fissette K, Fraga AG, Lee R, Mahrous E, Small PLC, Stragier P, Torrado E, Van Aerde A, Silva MT, Pedrosa J. 2008. First cultivation and characterization of *Mycobacterium ulcerans* from the environment. PLoS Negl Trop Dis 2:e178. doi:10.1371/journal.pntd.0000178. 18365032 PMC2268003

[4] Fyfe JAM, Lavender CJ, Johnson PDR, Globan M, Sievers A, Azuolas J, Stinear TP. 2007. Development and application of two multiplex real-time PCR assays for the detection of *Mycobacterium ulcerans* in clinical and environmental samples. Appl Environ Microbiol 73:4733–4740. doi:10.1128/AEM.02971-06. 17526786 PMC1951036

[5] Hammoudi N, Saad J, Drancourt M. 2020. The diversity of mycolactone-producing mycobacteria. Microb Pathog 149:104362. doi:10.1016/j.micpath.2020.104362. 32702376

[6] Buultjens AH, Vandelannoote K, Meehan CJ, Eddyani M, de Jong BC, Fyfe JAM, Globan M, Tobias NJ, Porter JL, Tomita T, Tay EL, Seemann T, Howden BP, Johnson PDR, Stinear TP. 2018. Comparative genomics shows that *Mycobacterium ulcerans* migration and expansion preceded the rise of Buruli ulcer in Southeastern Australia. Appl Environ Microbiol 84:e02612-17. doi:10.1128/AEM.02612-17. 29439984 PMC5881063

[7] Bankevich A, Nurk S, Antipov D, Gurevich AA, Dvorkin M, Kulikov AS, Lesin VM, Nikolenko SI, Pham S, Prjibelski AD, Pyshkin AV, Sirotkin AV, Vyahhi N, Tesler G, Alekseyev MA, Pevzner PA. 2012. SPAdes: a new genome assembly algorithm and its applications to single-cell sequencing. J Comput Biol 19:455–477. doi:10.1089/cmb.2012.0021. 22506599 PMC3342519

[8] Loftus MJ, Tay EL, Globan M, Lavender CJ, Crouch SR, Johnson PDR, Fyfe JAM. 2018. Epidemiology of Buruli ulcer infections, Victoria, Australia, 2011–2016. Emerg Infect Dis 24:1988–1997. doi:10.3201/eid2411.171593. 30334704 PMC6199991

[9] Fishbein S, van Wyk N, Warren RM, Sampson SL. 2015. Phylogeny to function: PE/PPE protein evolution and impact on *Mycobacterium tuberculosis* pathogenicity: evolution of PE/PPE-associated virulence. Mol Microbiol 96:901–916. doi:10.1111/mmi.12981. 25727695

[10] Seemann T. 2014. Prokka: rapid prokaryotic genome annotation. Bioinformatics 30:2068–2069. doi:10.1093/bioinformatics/btu153. 24642063

[11] Page AJ, Cummins CA, Hunt M, Wong VK, Reuter S, Holden MTG, Fookes M, Falush D, Keane JA, Parkhill J. 2015. Roary: rapid large-scale prokaryote pan genome analysis. Bioinformatics 31:3691–3693. doi:10.1093/bioinformatics/btv421. 26198102 PMC4817141

[12] Gouy M, Guindon S, Gascuel O. 2010. SeaView version 4: a multiplatform graphical user interface for sequence alignment and phylogenetic tree building. Mol Biol Evol 27:221–224. doi:10.1093/molbev/msp259. 19854763

[13] Saad J, Combe M, Hammoudi N, Couppié P, Blaizot R, Jedir F, Gozlan RE, Drancourt M, Bouam A. 2019. Whole-genome sequence of *Mycobacterium ulcerans* CSURP7741, a French Guianan clinical isolate. Microbiol Resour Announc 8:e00215-19. doi:10.1128/MRA.00215-19. 31320424 PMC6639603

