# Supplementary material for: PPE Barcoding Identifies Biclonal Mycobacterium ulcerans Buruli Ulcer, Côte d’Ivoire
Source: Microbiol Spectr. 2023 May 24;11(3):e00342-23. doi: 10.1128/spectrum.00342-23 (PMC10269924; doi:10.1128/spectrum.00342-23)
Supplement: Supplemental file 1 — Captions for Tables S1 to S3. Download spectrum.00342-23-s0002.pdf, PDF file, 0.4 MB [file spectrum.00342-23-s0002.pdf]

SUPPORTING INFORMATION.

Table S1: Clinical information of 385 study strains like geographic isolation and host.

Table S2: SNPs distance between 385 genomes study strains showed the distribution values of SNPs between different announced taxa of *M. ulcerans* and *M. marinum*.

Table S3: All mutations or deletions detected in the common region sequence of *PPE* (proline-proline-glutamate; GenBank *PPE* gene ID for *Mycobacterium ulcerans* Agy99: ABL03116.1) gene based on announced taxa.

REFERENCES

1. Zingue D, Bouam A, Tian RBD, Drancourt M. 2018. Buruli Ulcer, a Prototype for Ecosystem-Related Infection, Caused by *Mycobacterium ulcerans*. Clin Microbiol Rev 31:e00045-17.

2. Van Leuvenhaege C, Vandelannoote K, Affolabi D, Portaels F, Sopoh G, de Jong BC, Eddyani M, Meehan CJ. 2017. Bacterial diversity in Buruli ulcer skin lesions: Challenges in the clinical microbiome analysis of a skin disease. PLoS ONE 12:e0181994.

3. Portaels F, Meyers WM, Ablordey A, Castro AG, Chemlal K, de Rijk P, Elsen P, Fissette K, Fraga AG, Lee R, Mahrous E, Small PLC, Stragier P, Torrado E, Van Aerde A, Silva MT, Pedrosa J. 2008. First Cultivation and Characterization of *Mycobacterium ulcerans* from the Environment. PLoS Neglected Tropical Diseases 2:e178.

4. Fyfe JAM, Lavender CJ, Johnson PDR, Globan M, Sievers A, Azuolas J, Stinear TP. 2007. Development and Application of Two Multiplex Real-Time PCR Assays for the Detection of *Mycobacterium ulcerans* in Clinical and Environmental Samples. Appl Environ Microbiol 73:4733–4740.

5. Hammoudi N, Saad J, Drancourt M. 2020. The diversity of mycolactone-producing mycobacteria. Microbial Pathogenesis 149:104362.

6. Buultjens AH, Vandelannoote K, Meehan CJ, Eddyani M, de Jong BC, Fyfe JAM, Globan M, Tobias NJ, Porter JL, Tomita T, Tay EL, Seemann T, Howden BP, Johnson PDR, Stinear TP. 2018. Comparative Genomics Shows That *Mycobacterium ulcerans* Migration and Expansion Preceded the Rise of Buruli Ulcer in Southeastern Australia. Appl Environ Microbiol 84:e02612-17.

7. Bankevich A, Nurk S, Antipov D, Gurevich AA, Dvorkin M, Kulikov AS, Lesin VM, Nikolenko SI, Pham S, Prjibelski AD, Pyshkin AV, Sirotkin AV, Vyahhi N, Tesler G, Alekseyev

MA, Pevzner PA. 2012. SPAdes: A New Genome Assembly Algorithm and Its Applications to Single-Cell Sequencing. Journal of Computational Biology 19:455–477.

8. Loftus MJ, Tay EL, Globan M, Lavender CJ, Crouch SR, Johnson PDR, Fyfe JAM. 2018. Epidemiology of Buruli Ulcer Infections, Victoria, Australia, 2011–2016. Emerg Infect Dis 24:1988–1997.

9. Fishbein S, van Wyk N, Warren RM, Sampson SL. 2015. Phylogeny to function: PE/PPE protein evolution and impact on *M ycobacterium tuberculosis* pathogenicity: Evolution of PE/PPE-associated virulence. Molecular Microbiology 96:901–916.

10. Seemann T. 2014. Prokka: rapid prokaryotic genome annotation. Bioinformatics 30:2068–2069.

11. Page AJ, Cummins CA, Hunt M, Wong VK, Reuter S, Holden MTG, Fookes M, Falush D, Keane JA, Parkhill J. 2015. Roary: rapid large-scale prokaryote pan genome analysis. Bioinformatics 31:3691–3693.

12. Gouy M, Guindon S, Gascuel O. 2010. SeaView Version 4: A Multiplatform Graphical User Interface for Sequence Alignment and Phylogenetic Tree Building. Molecular Biology and Evolution 27:221–224.

13. Saad J, Combe M, Hammoudi N, Couppié P, Blaizot R, Jedir F, Gozlan RE, Drancourt M, Bouam A. 2019. Whole-Genome Sequence of *Mycobacterium ulcerans* CSURP7741, a French Guianan Clinical Isolate. Microbiol Resour Announc 8:e00215-19.

14. Vandelannoote K, Meehan CJ, Eddyani M, Affolabi D, Phanzu DM, Eyangoh S, Jordaens K, Portaels F, Mangas K, Seemann T, Marsollier L, Marion E, Chauty A, Landier J, Fontanet A, Leirs H, Stinear TP, de Jong BC. 2017. Multiple introductions and recent spread of the emerging human pathogen *Mycobacterium ulcerans* across Africa. Genome Biol Evol evx003.

15. Bahadoran P, Hammoudi N, Gaudart A, Saad J, Di Filippo Y, Drancourt M, Ruimy R. 2021. Case Report: A New *Mycobacterium ulcerans* Genotype Causing Buruli Ulcer in Côte d'Ivoire. The American Journal of Tropical Medicine and Hygiene 104:1782–1783.
